# Implicit Perceptions of Closeness From the Direct Eye Gaze

**DOI:** 10.3389/fpsyg.2018.02673

**Published:** 2019-01-07

**Authors:** Mengmeng Cui, Minghao Zhu, Xiaomin Lu, Lei Zhu

**Affiliations:** ^1^Department of Psychology, Fudan University, Shanghai, China; ^2^Department of Consumer Science, Purdue University, West Lafayette, IN, United States

**Keywords:** IAT, direct gaze, closeness, eye gaze direction, implicit association

## Abstract

Eye gaze plays an important role during social interaction. Specifically, different eye gaze directions may send different functional messages to the observers, who have the capacity to automatically interpret these signals. In the present study, we used the implicit association test (IAT) to investigate whether direct eye gaze sends a functional, automatically perceived signal about non-target interpersonal closeness. Results suggest that the direct gaze strongly signals close relationship, and this association cannot be accounted for by positive valence. The findings suggest that the direct gaze may function to uniquely communicate a generalized closeness without orientation. Discussion focuses on the implications of these findings for social functions of direct gaze during interpersonal interaction and the automatic nature of such associations.

## Implicit Perceptions of Closeness From the Direct Eye Gaze

Eye gaze plays an important role during social interaction, such as regulation of interaction, facilitation of communication, and expression of intimacy and social control (Kleinke, 1986). There is evidence that people interpret eye gaze cues available from others’ faces to denote social closeness between themselves and the target persons. However, it is not clear whether or not they can acquire a non-target (non-oriented) feeling of closeness from direct gaze. Information about such generalized, spontaneously perceived closeness when viewing direct gaze would be useful to understanding the social interaction processes. Thus, the present study aims to investigate its social functions (i.e., whether direct eye gazes convey information about non-target closeness).

Eye gaze is one of the ostensive social signals which indicates something of importance to be communicated (Frith, 2008). Eye gaze not only conveys rich important information about another’s focus of attention, but also implies his or her future intentions and actions (Baron-Cohen, 1995). Knowing whether another person directly gazes at you is crucial because it leads people to generate a corresponding response (e.g., smile, run away, or search for further communication).

From an evolutionary perspective, people have evolved to understand the intentions of others by using eye gaze cues. Thus, the ability to detect gaze direction is present from early infancy. Even newborn infants attend more to the eyes than other parts of the face (Morton and Johnson, 1991). From several months old, infants can discriminate direct from averted gaze (Vecera and Johnson, 1995; Farroni et al., 2000). By the age of 9 to 18 months, infants can infer the target direction from adults’ eye gaze (Phillips et al., 1992).

For adults, gaze direction serves more cognitive functions (Kleinke, 1986). Direct gaze potentiates detection of faces (Hood et al., 2003; Senju and Hasegawa, 2005), enhances facial gender categorization, and facilitates access to gender-related semantic information (Macrae et al., 2002). Also, direct gaze promotes implicit memory for faces (Mason et al., 2004; Vuilleumier et al., 2005). On the other hand, averted gaze shifts visual attention to the area around the gaze direction (Friesen and Kingstone, 1998; Driver et al., 1999; Hietanen, 1999).

An equally long-standing researched line of inquiry has examined the social function of eye gaze (Kleinke, 1986; Baron-Cohen, 1994, 1995; Emery, 2000). In this vein, eye gaze has been assumed to serve a social function; for example, implying the mental states of others (Baron-Cohen, 1994, 1995; Emery, 2000). Indeed, the activation of “mentalizing” areas when viewing another’s eye gaze, both direct and averted gaze (Calder et al., 2002; Kampe et al., 2003; Wicker et al., 2003) is consistent with the claim that eye gaze serves social functions. Further, studies about dyadic social interaction also suggested that increased eye contact led to more positive interactions (Hessels et al., 2017). Eye contact during live social interaction increased infants’ oscillatory brain activity (Hoehl et al., 2014). Also, eye contact inhibited aggression in police-citizen interaction (Boyanowsky and Griffiths, 1982).

The positive impact of direct gaze on social interaction may have to do with the linkage between direct gaze and perceived closeness. Several researchers have suggested that direct eye gaze conveyed information about closeness. Adams and Kleck (2003) proposed that gaze direction signaled the expresser’s approach-avoidance behavioral tendencies. Direct gaze is related to approach behavior and averted gaze is associated with avoidance. It is demonstrated that people who looked at the observer were judged to be more approachable than those who looked away from the observer (Willis et al., 2011). More direct support comes from the immediacy model of social intimacy (Mehrabian, 1967) which claimed that increased eye contact could enhance psychological closeness. Following this line of thought, in a communication simulation experiment, participants were asked to deliver a personally revealing monolog to a same-sex listener whose gaze either directed to, or averted from, them. Speakers rated their communications as more intimate in the direct gaze condition than in the averted gaze condition (Ellsworth and Ross, 1975). Further, in our previous study, participants were required to rate the closeness between themselves and the target persons by the Inclusion of Other in the Self (IOS) scale (Zhou et al., 2018). Similarly, the target persons were judged to be closer when looking at the observers rather than looking away from the observers.

In sum, there is evidence that people interpret eye gaze cues available from others’ faces to denote social closeness between themselves and the target persons. However, it is not clear whether or not they can acquire a non-target (non-oriented) feeling of closeness from direct gaze. Information about such generalized, spontaneously perceived closeness when viewing direct gaze would be useful to understand the social interaction processes.

One way to test whether children interpret direct gaze as closeness (not limited to the target person) is to adopt the implicit association test (IAT; Greenwald and Banaji, 1995). In the two experiments, participants completed an IAT that paired photographs of different gaze directions with words (Experiment 1) or pictures (Experiment 2) representing close or distant relationship, and we compared the speed of their responses to presumed congruent versus incongruent pairings. The participants were college students. The same faces were used for two conditions (direct and averted gaze) to avoid participants associating the perceived closeness to a target person. In Experiment 1, we used words (e.g., friend or enemy) to indicate closeness. In Experiment 2, we used two pictures illustrating two persons at a different distance (close or distant) between them to represent close- and distant-relationship. Thus, for Experiment 1, the non-target closeness means the closeness between everybody around the observers and themselves. For Experiment 2, the non-target closeness means the closeness between two other persons. If participants were faster to respond to the presumed congruent pairing (direct gaze with close relationship and averted gaze with distant relationship) than to the incongruent pairing (direct gaze with distant relationship and averted gaze with close relationship), it would indicate a stronger implicit association between direct gaze and close rather than distant relationship. Such findings would indicate that the process of direct gaze in signaling closeness occurs without intention and cannot be controlled because participants do not make a conscious effort to do so. Information about such generalized, spontaneously perceived closeness when viewing direct gaze would be very important, because it can sway an individual’s social interaction and his or her understanding of the world. For Experiment 1, we questioned whether the perceived closeness from the experience of eye contact alone could transfer to everyone around the observers and enhance their feeling of accessible social support. On the other hand, for Experiment 2, we questioned whether direct gaze from a target person could sway participants’ understanding of the closeness between two strangers and lead them to the conclusion that the human world is getting “warmer and warmer.”

## Experiment 1

For Experiment 1, we predicted that participants would be faster to respond to the presumed congruent pairing (direct gaze with close relationship and averted gaze with distant relationship) than to the incongruent pairing (direct gaze with distant relationship and averted gaze with close relationship).

### Methods

#### Participants

We ran a power analysis using G^∗^Power 3.1 (Faul et al., 2007), with an estimated power of 0.8, a medium effect size of 0.52 (d) (Borenstein et al., 1990). The power analysis revealed that 32 participants needed to be tested. Thirty-two volunteers from the university community with normal or corrected-to-normal vision (16 males, aged from 18 to 25, *M* = 19.94, *SD* = 1.27) participated in this experiment. All the participants were given a small gift for their participation.

#### Materials

##### Words

Ten two-character Chinese adjectives were used as materials. Half of the words represent close relationship and the remaining half represent distant relationship (Appendix). We derived these words by obtaining synonyms or antonyms for “close relationship” and “distant relationship” from a synonyms and antonyms dictionary.

##### Eye gazes

**FIGURE 1:**
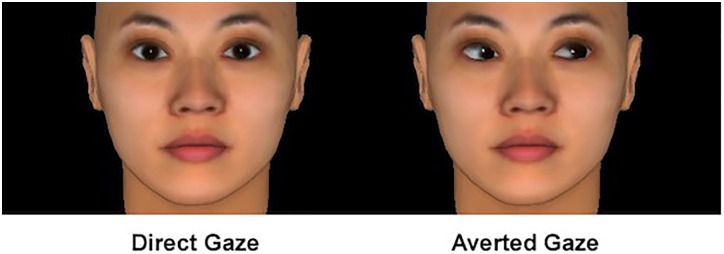
Samples gaze photos in Experiment 1.

We used FaceGen software^1^ to generate ten photographs of Southeast Asian faces posing with neutral expressions, of which five were female and five male (Figure 1). However, FaceGen software cannot generate faces of people in a specific country, such as China. It can only generate faces of people in a broader area. Since the participants were Chinese, only Southeast Asian faces were used. Only same-sex faces were presented to the participants because gazes by the opposite sex are often confused with many other factors, such as mate selection preferences or romance (Feingold, 1991). Another 10 photographs of the same faces were generated by changing the gaze direction parameter in FaceGen to “look at the right.” Thus, for each participant the same five faces were presented for two conditions (direct gaze vs. averted gaze). Such manipulation can avoid participants associating the perceived closeness to a target person.

#### Procedures

Participants were required to complete a standard IAT pairing different gaze direction with words representing either close or distant relationship. They categorized the faces as either direct or averted gaze and the words as close- or distant-relationship, by pressing one of two keys (“E” or “I”) as accurately and fast as possible. There were seven blocks: (1) Target discrimination task 1, which had 20 trials and required participants to categorize the faces as either direct or averted gaze; (2) Attribute discrimination task, which had 20 trials and required participants to categorize the words as close- or distant-relationship; (3) Practice of the first combined task, which had 20 trials; (4) Test of the first combined task, which had 40 trials; (5) Target discrimination task 2, which was similar to Target discrimination task 1 except that the parings of eye gaze and response keys were reversed; (6) Practice of the second combined task, which had 20 trials; (7) Test of the second combined task, which had 40 trials. In one combined task, the direct-gaze photos and the close-relationship words shared a key, and the averted-gaze photos and the distant-relationship words shared a key. In the other combined task, these pairings were reversed (i.e., direct-gaze and distant-relationship shared a key, and averted-gaze and close-relationship shared a key). The order of these pairings was counterbalanced between participants.

#### Results and Discussion

We calculated implicit associations according to the scoring algorithm proposed by Greenwald et al. (2003). First, responses that were longer than 10 s were excluded from analysis. Second, there were no participants for whom more than 10% of trials were shorter than 300 ms. Third, each error latency was replaced by an error penalty computed as block mean of correct response latencies plus twice the standard deviation of correct response latencies in that block. Then, we calculated difference scores separately for practice and test blocks by dividing the difference between the error-adjusted mean reaction times of two combined tasks by their overall standard deviations for the two tasks. Last, the overall difference score (d score) was computed for each individual by averaging two difference scores, indicating the strength and direction of participants’ implicit association. One-sample *t*-tests were carried out to determine whether these d scores differed significantly from zero. Thus, mean d scores (Greenwald et al., 2003) across participants, one-sample *t*-test statistics, and Cohen’s d*s* that indicate the overall sample effect sizes were reported for the two experiments.

**FIGURE 2:**
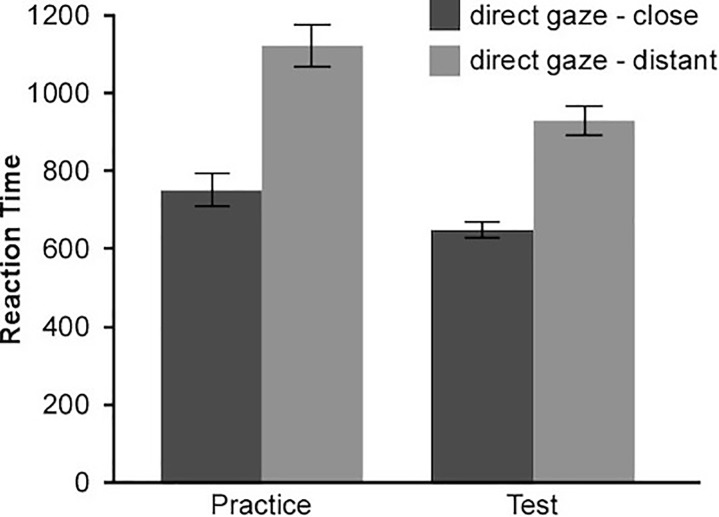
Mean reaction times(ms) for two combined tasks (Experiment 1). Results showed that direct gaze was implicitly associated with closeness. Error bars indicates ± standard errors.

The mean reaction times of two combined tasks were displayed in Figure 2. One-sample *t*-test revealed that participants showed a strong implicit association between direct gaze and closeness, *d* = 0.83, *t*(31) = 9.51, *p* < 0.001, Cohen’s *d* = 1.68. They responded faster when pairing the direct-gaze with the close-relationship and the averted-gaze with the distant-relationship than when pairing the direct-gaze with the distant-relationship and the averted-gaze with the close-relationship. Although participants were required to respond at the same quick rate for each trail, the difference between two combined tasks also appeared. Further, the d score of male and female participants did not differ from each other *t*(30) = 0.32, *p* = 0.755.

This finding suggests that the direct-gaze is more strongly associated with close-relationship than the averted-gaze, and that the averted-gaze is more strongly associated with distant-relationship than the direct-gaze at an implicit level. The direct-gaze might derive an implicit non-target perception of closeness.

## Experiment 2

However, it is too early to draw a conclusion. Given that the words representing close-relationship (e.g., friend) are more positive than the words representing distant-relationship (e.g., enemy), it is possible that the association between direct-gaze and positive valence accounts for the difference of two combined tasks, because as words representing close-relationship, direct gaze might also be more positive than averted gaze. Thus, in Experiment 2, instead of words, we used two pictures to indicate closeness. It is predicted that participants would be faster to respond to the presumed congruent pairing (direct gaze with close relationship and averted gaze with distant relationship) than to the incongruent pairing (direct gaze with distant relationship and averted gaze with close relationship).

### Methods

#### Participants

We ran a power analysis using G^∗^Power 3.1 (Faul et al., 2007), with an estimated power of 0.8, a medium effect size of 0.52 (*d*) (Borenstein et al., 1990). The power analysis revealed that 32 participants needed to be tested. Thirty-two volunteers from the university community with normal or corrected-to-normal vision (16 males, aged from 19 to 23, *M* = 20.94, *SD* = 1.05) participated in this experiment. All the participants were given a small gift for their participation.

#### Materials

**FIGURE 3:**
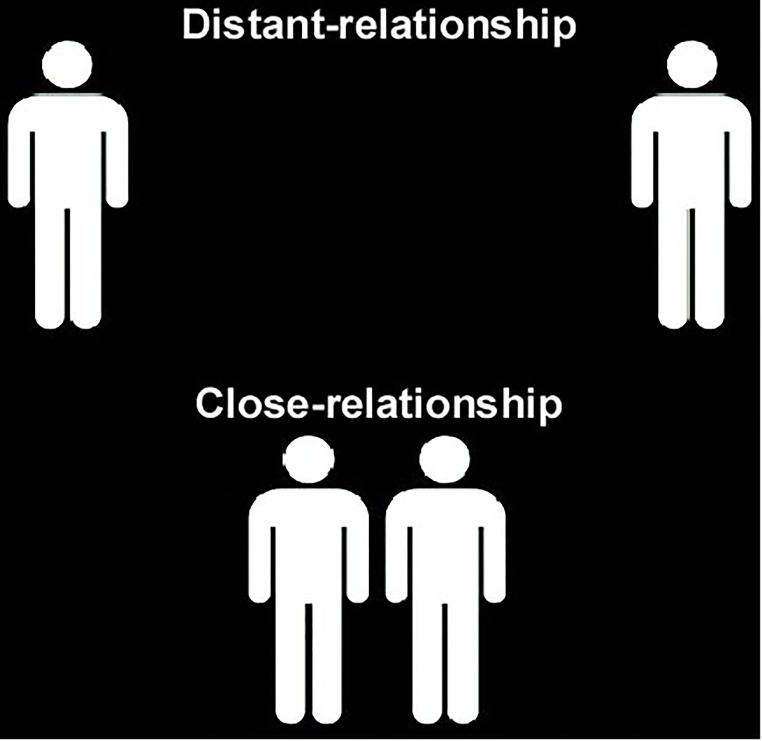
Two pictures representing close- and distant-relationship in experiment 2.

The materials were similar to those used in Experiment 1, except that two pictures were used instead of words to represent close- and distant-relationship (Figure 3). The pictures illustrated two persons at a different distance (close or distant) between them to represent close- and distant-relationship. Ten adults from the university community [8 males, average age 20.60 (*SD* = 0.52)] who were not included in the formal experiment were recruited to rate the valence of pictures on a 7-point Likert scale, with 1 indicating extremely negative and 7 indicating extremely positive. The presentation order of the two pictures was counterbalanced between participants. The difference of ratings for two pictures was not statistically reliable [*t*(9) = 0.43, *p* = 0.678]. Also, all 10 adults were required to choose which picture indicated the close-relationship. All of them were correct.

#### Procedures

The procedures were identical to Experiment 1.

#### Results and Discussion

**FIGURE 4:**
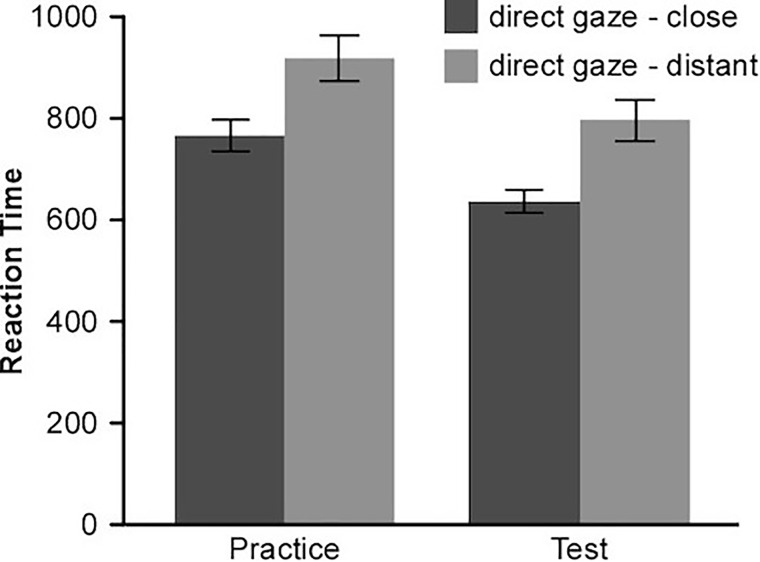
Mean reaction times(ms) for two combined tasks (Experiment 2). Results showed that direct gaze was implicitly associated with closeness. Error bars indicates ± standard errors.

As in Experiment 1, we calculated implicit associations according to the scoring algorithm proposed by Greenwald et al. (2003). The mean reaction times of two combined tasks are displayed in Figure 4. One-sample *t*-test revealed that participants showed a strong implicit association between direct gaze and closeness, *d* = 0.39, *t*(31) = 3.63, *p* = 0.001, Cohen’s *d* = 0.64. They responded faster when pairing the direct-gaze with the close-relationship and the averted-gaze with the distant-relationship than pairing the direct-gaze with the distant-relationship and the averted-gaze with the close-relationship. Although participants were required to respond at the same quick rate for each trial, the difference between two combined tasks also appeared. Further, the d score of male and female participants did not differ from each other *t*(30) = 1.47, *p* = 0.151.

This finding suggests that the direct-gaze is more strongly associated with close-relationship than the averted-gaze, and that the averted-gaze is more strongly associated with distant-relationship than the direct-gaze at an implicit level. The direct-gaze might derive an implicit non-target perception of closeness.

## Discussion

The present study provides evidence for an implicit association between the direct gaze and the concept of closeness. The association between direct gaze and close relationship emerged when it was compared with averted gaze. This association also emerged when using two pictures without valence difference to substitute relationship words, suggesting that the association cannot be accounted for by positive valence (i.e., both direct-gaze and close-relationship are positive).

Although there is evidence that people interpret eye gaze cues available from others’ faces to denote social closeness between themselves and the target persons (Ellsworth and Ross, 1975; Willis et al., 2011; Zhou et al., 2018), it is not clear whether or not they can acquire a non-target (non-oriented) feeling of closeness from direct gaze. Expanding on these previous studies, the presented study measured the association between direct gaze and non-target closeness by IAT and found that the direct eye gaze is a fairly specific signal of non-target closeness. The present findings are very important because information about such generalized, spontaneously perceived closeness when viewing direct gaze could sway an individual’s social interaction and his or her understanding of the world. The perceived closeness from an experience of eye contact alone might transfer to everyone around the observers and enhance their feeling of accessible social support. On the other hand, direct gaze seems to be a signal of a warm world. Direct gaze from a target person could sway participants’ understanding of the relationship between others (two persons in the pictures of Experiment 2) and lead them to the conclusion that the human world is getting “warmer and warmer.”

More broadly, we argue that enhanced closeness by direct gaze might be an important mechanism underlying studies about dyadic social interaction. It is demonstrated that increased eye contact led to more positive interactions (Hessels et al., 2017). For example, eye contact inhibited aggression in police-citizen interaction (Boyanowsky and Griffiths, 1982). The reduced aggression might be caused by a feeling of closeness and accessible social support obtained from eye contact. Adams and Kleck (2003) used a similar mechanism to explain the relation between eye gaze and different facial expressions. They presented angry, joyful, fearful, and sad faces with direct and averted gaze and found that participants identified anger and joy expressions faster for faces with direct gaze than with averted gaze. However, they identified fear and sad expressions faster for faces with averted gaze than with direct gaze. They explained that this was because both direct gaze and anger or joy signaled approaching and both averted gaze and fear or sadness signaled avoidance. The approaching-avoidance explanation is similar to the concept of closeness.

However, direct gaze does not convey information about closeness all the time. Eye contact from outgroup or higher status groups might not be viewed positively or as a warm sign of connection (Frieze and Ramsey, 1976; Ridgeway, 1987; Allison et al., 2000; Todorov et al., 2008; Foulsham et al., 2010; Holland et al., 2017). The present findings showed that direct gaze from a stranger conveys information about closeness. Further studies might focus on demonstrating the effect of eye gaze on different individuals.

It should be noted that the materials used to denote closeness were different for the two experiments. Experiment 1 used words and Experiment 2 used pictures. Upon closer examination, it is obvious that the relationship indicated by words is self-oriented and the relationship indicated by pictures is other-oriented. Thus, the closeness signaled by direct gaze might not be limited to the observers themselves but be applied to every person in the world.

Moreover, the current results demonstrate that closeness perceptions of the direct gaze are unelaborated and automatic. This is the first research to suggest that our ability to rapidly and involuntarily assess interpersonal closeness may be due, in part, merely by an eye gaze from an unrelated person. In the two IAT experiments, although participants were required to respond at the same quick rate to all stimuli when viewing different relationship words or pictures and faces of different eye gaze, they still had markedly more difficulty inhibiting to associate direct gaze with close-relationship than inhibiting the associations between direct gaze and distant-relationship. It is implied that interpreting processes regarding the closeness triggered by the direct gaze cannot be consciously controlled. Thus, the present findings provide insight into how closeness is automatically perceived in our everyday social interactions. Individuals may approach any others implicitly, on the basis of eye gaze from an unrelated person, even when they wish to avoid doing so. Such gained closeness with any social group member is an important part of interpersonal interaction.

The present study demonstrated that IAT could be applied to examine the social function of eye gaze. This is a novel application of the IAT, which might be used for future research on other body signals, given the strength of the effects found in the present study. For example, it is well known that stretching out arms or smile expression also signal closeness. IAT could be used to determine whether the implicit associations are equally strong for all three facial or body signals. It could also be used to examine how other facial signals influence the way individuals perceive the messages sent by a particular eye gaze direction. For example, does the association between direct gaze and closeness depend on whether the target person is happy or angry? N’Diaye et al. (2009) implied that direct gaze with an angry expression signaled aggressiveness. Willis et al. (2011) found that compared to averted gaze with an angry expression, direct gaze with an angry expression was perceived as less approachable.

## Conclusion

In conclusion, expanding on previous studies demonstrating that direct gaze increased target-oriented closeness, the presented study found that the direct eye gaze conveyed information about non-target closeness between the observers and everyone around them or between two other persons. These findings are very important because information about such generalized, spontaneously perceived closeness when viewing direct gaze could sway an individual’s social interaction and his or her understanding of the world. The perceived closeness from an experience of eye contact alone might transfer to everyone around the observers and enhance their feeling of accessible social support. On the other hand, direct gaze seems to be a signal of a warm world. Direct gaze from a target person could sway participants’ understanding of the relationship between others and lead them to the conclusion that the human world is getting “warmer and warmer.”

## Ethics Statement

The data was collected during 2013-2014. We cannot find an ethics committee that could approve the study at that time.

## Author Contributions

MC wrote the paper. MZ and XL carried out the experiments. LZ designed the study and wrote the paper.

## Conflict of Interest Statement

The authors declare that the research was conducted in the absence of any commercial or financial relationships that could be construed as a potential conflict of interest.

## Footnotes

1 https://facegen.com/index.htm

## Appendix

**Table:** Five words representing close relationship.

Adjective	In English
	Friend
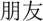
	Acquaintance
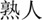
	Family
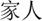
	Lover
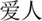
	Wife
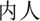

**Table:** Five words representing distant relationship.

Adjective	In English
	Enemy
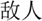
	Stranger
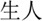
	Passer-by
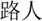
	Foe
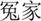
	Outsider
